# Supplementary figures and images for: Detection of Lysyl Oxidase Activity in Tumor Extracellular Matrix Using Peptide-Functionalized Gold Nanoprobes
Source: Cancers (Basel). 2021 Sep 8;13(18):4523. doi: 10.3390/cancers13184523 (PMC8471099; doi:10.3390/cancers13184523)

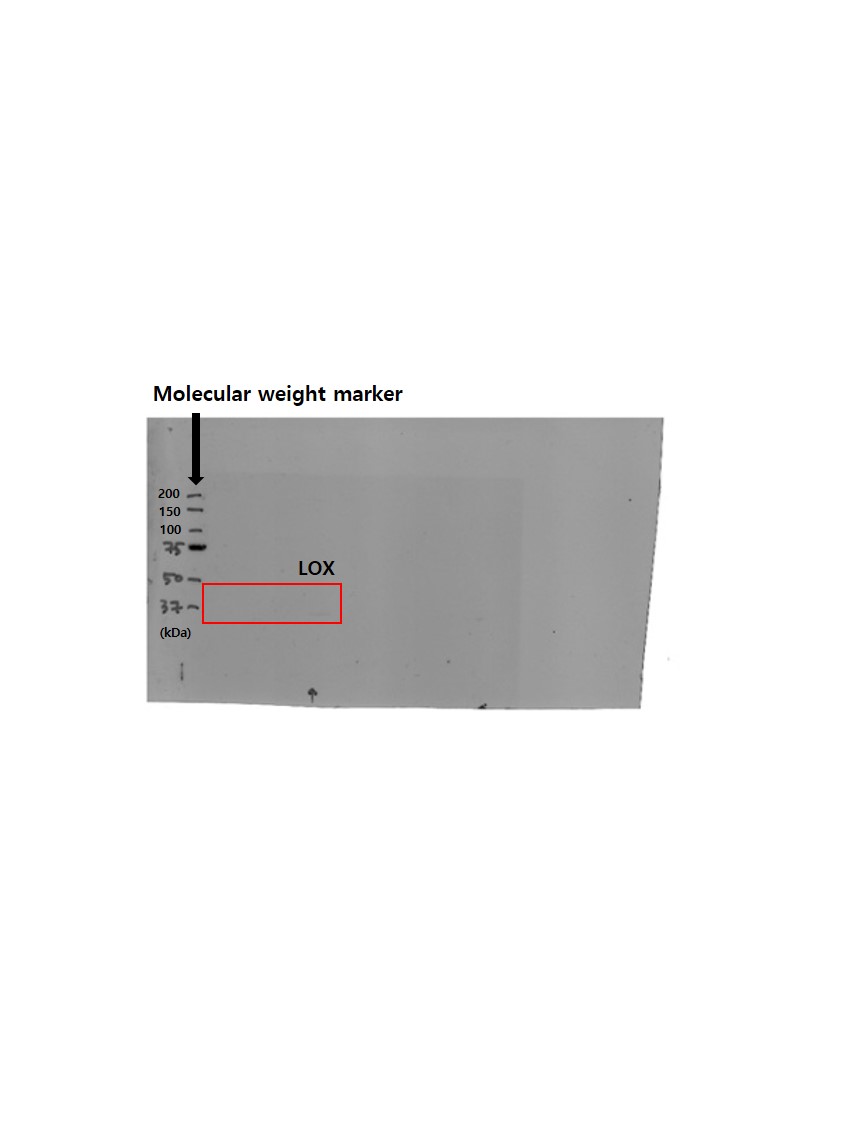

Supplement: Supplementary file 1 [file cancers-13-04523-s001.zip › cancers-1315721-Supplementry Materials/FIgure 5d_LOX.jpg]

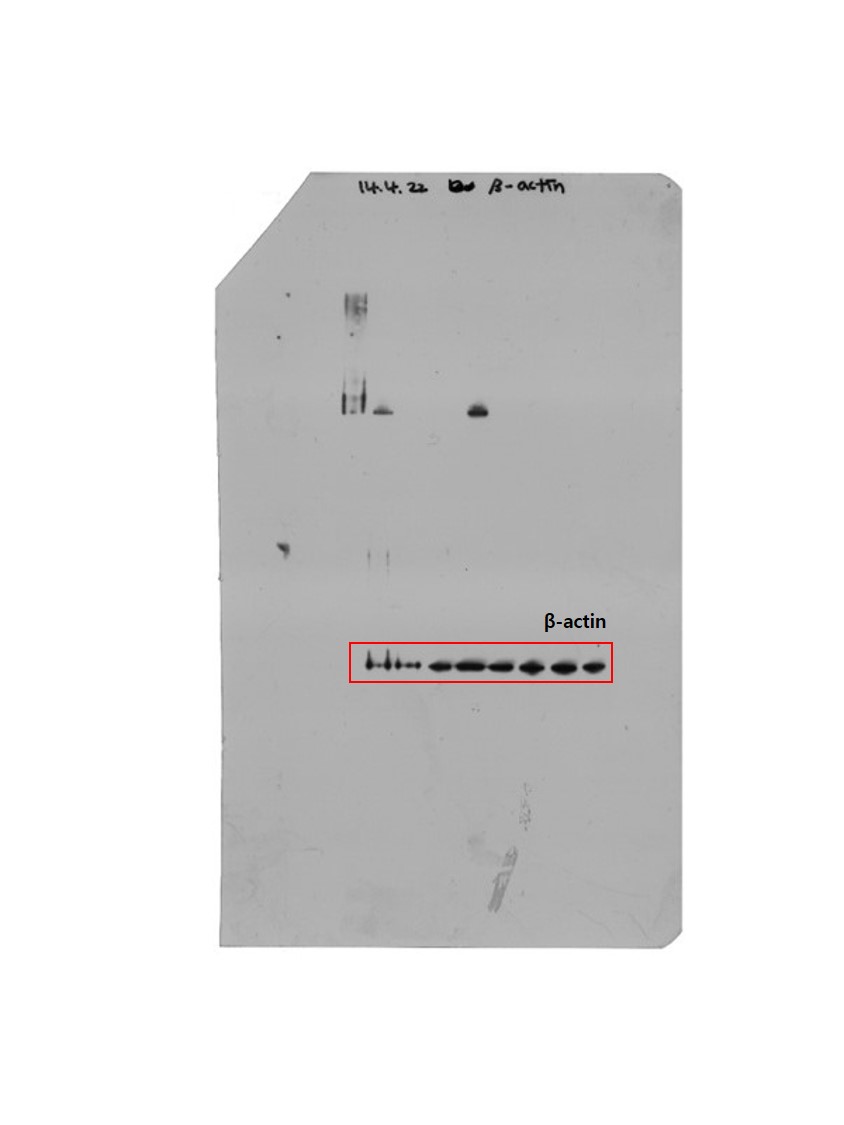

Supplement: Supplementary file 1 [file cancers-13-04523-s001.zip › cancers-1315721-Supplementry Materials/Figure 6b_Beta-actin.jpg]

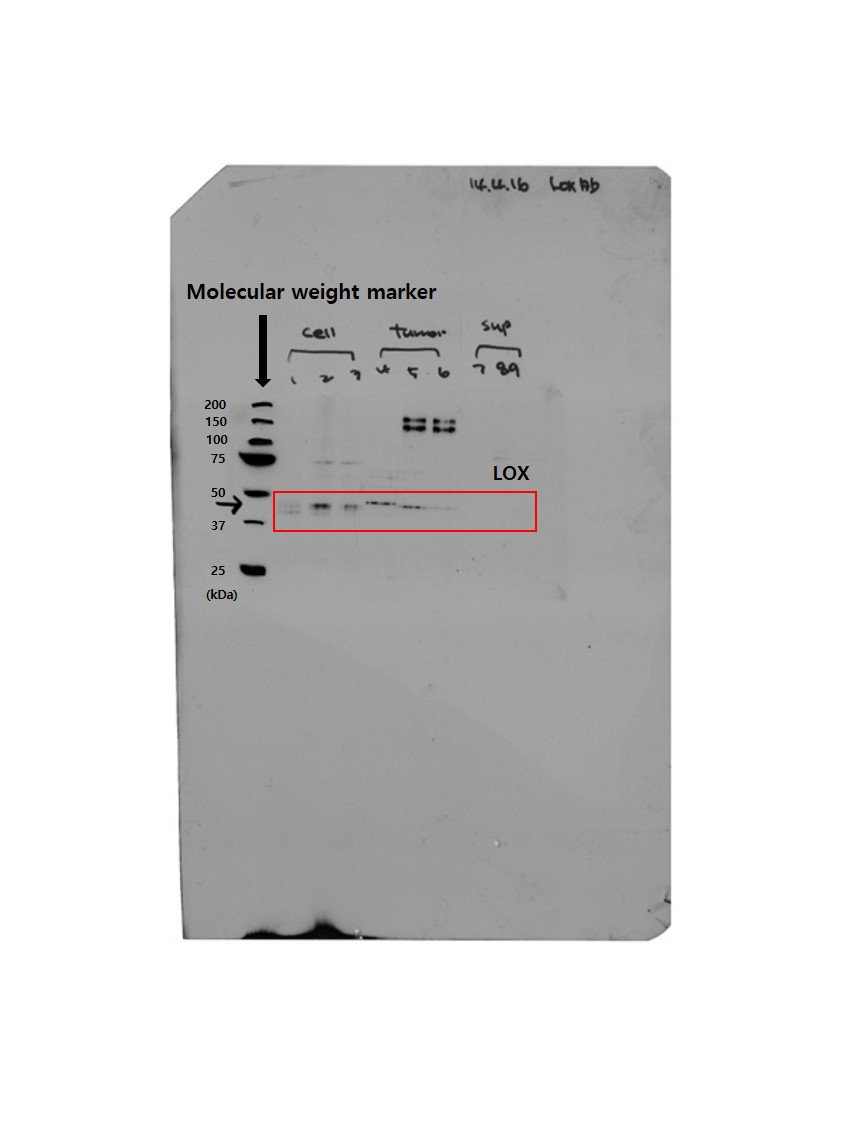

Supplement: Supplementary file 1 [file cancers-13-04523-s001.zip › cancers-1315721-Supplementry Materials/Figure 6b_LOX.jpg]
